# Bioactive Polysaccharides from *Hericium erinaceus*: Extraction, Structure, Bioactivities, and Applications

**DOI:** 10.3390/molecules30081850

**Published:** 2025-04-20

**Authors:** Fangzhi Ge, Yan Chen, Binshuo Wang, Wenxin Zhou, Baoxiang Du, Lin Hou

**Affiliations:** 1 Marine Traditional Chinese Medicine Research Institute (Qingdao Academy of Traditional Chinese Medicine), Shandong University of Traditional Chinese Medicine, Qingdao 266112, China; gefangzhi@163.com (F.G.); cheny910@126.com (Y.C.); 2 School of Pharmacy, Shandong University of Traditional Chinese Medicine, Jinan 250355, China; 19862936202@163.com; 3 School of Health Sciences, Shandong University of Traditional Chinese Medicine, Jinan 250355, China; 18653187732@163.com

**Keywords:** *Hericium erinaceus* polysaccharide, purification, structural analysis, biological activity, toxicity, applications

## Abstract

*Hericium erinaceus*, an edible fungus belonging to the family Odontaceae, is predominantly found in Western Europe, North America, and East Asia. In China, it primarily thrives in the mountainous and forested regions in the northeast, north, and southwest. Historically, *Hericium erinaceus* has served as a medicinal and nutritional entity. Its mycelia and fruiting bodies are the products of its vegetative growth stage and reproductive growth stage, respectively. The principal active components are different *Hericium erinaceus* polysaccharides (HEPs), which are a group of polysaccharides primarily composed of galactose, glucose, and a small amount of mannose and fucose. An extremely small number of HEPs contain fructose, glucuronic acid, xylose, arabinose, and other components. The common extraction method employed is water extraction followed by alcohol precipitation. HEPs exhibit a diverse array of biological activities, including immune enhancement, anti-tumor effects, anti-inflammatory properties, antioxidant capabilities, and antiviral functions. This paper provides a comprehensive review of recent advancements in the extraction, separation, purification, structural analysis, biological activity, and toxicity assessments of HEPs. Additionally, it discusses the opportunities and challenges associated with scientific research and practical applications in this field.

## 1. Introduction

*Hericium erinaceus* (Bull.) Pers., a species within the genus *Hericium*, is predominantly found in broad-leaved forests and mixed forests comprising both coniferous and broad-leaved trees in the northern temperate zone, including regions such as Eastern Europe, Oceania, North America, and East Asia (e.g., Japan and China) [1,2]. The fruiting bodies of *Hericium erinaceus* (HEFBs) are commonly encountered in natural settings and can also be cultivated through artificial means. The mycelium of *Hericium erinaceus* (HEM) is typically generated via deep fermentation of *Hericium erinaceus* strains in artificial media. In recent decades, a significant number of metabolites have been isolated from HEFBs, including compounds such as sinapine, glucosinolates, glycoproteins, polysaccharides, and sterols [1,3].

**Figure 1:**
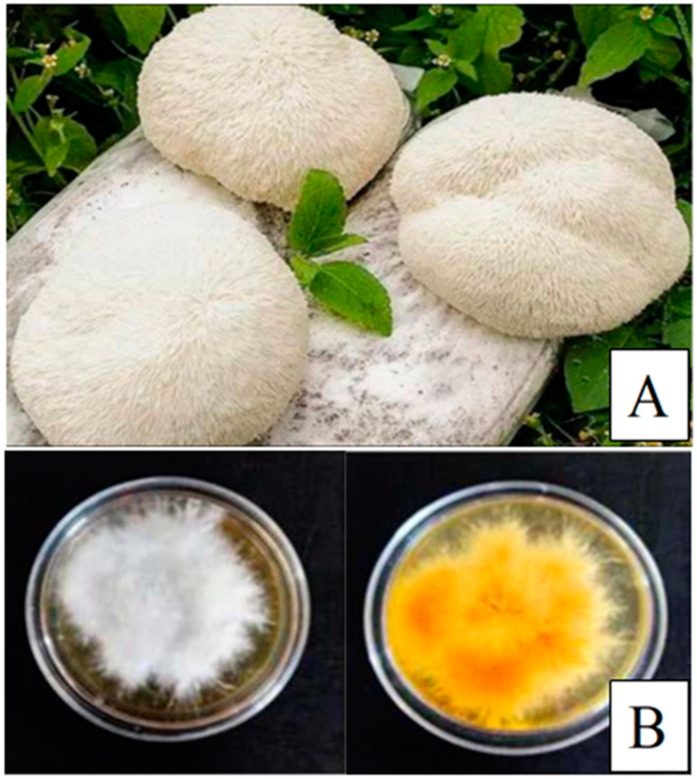
*Hericium erinaceus*. (**A**) The fruiting body of *Hericium erinaceus*. (**B**) The mycelium of *Hericium erinaceus*.

**Figure 2:**
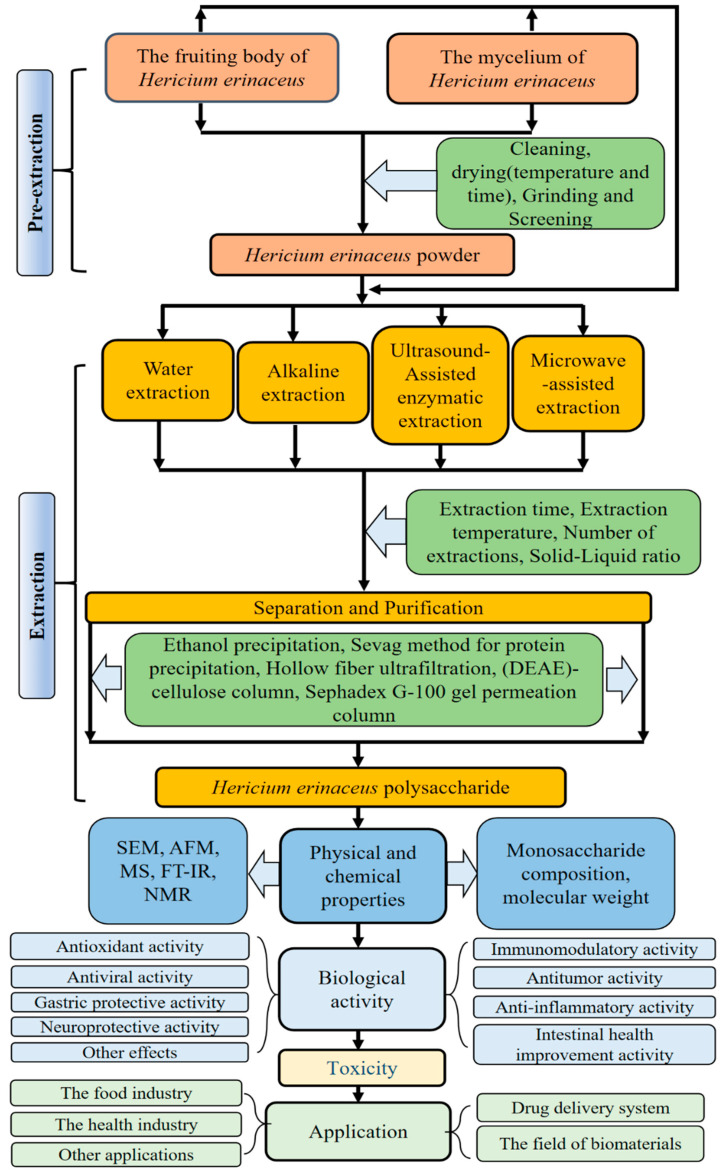
Schematic diagram of HEP’s pretreatment, extraction, separation, purification, physicochemical properties, structural characteristics, biological activity, and application, with scanning electron microscopy (SEM), atomic force microscopy (AFM), mass spectrometry (MS), Fourier transform-infrared spectroscopy (FT-IR), and nuclear magnetic resonance (NMR).

*Hericium erinaceus*, a rare edible fungus, has garnered significant attention in research and product development, particularly concerning its fruiting body and mycelium (Figure 1). *Hericium erinaceus* has a long history of use in traditional medicine, particularly within the frameworks of traditional healing practices. Ancient healers recognized its potential to promote cognitive and neurological health, often employing it to enhance memory and focus, while also observing its capacity to support overall brain function. Modern scientific inquiry has begun to unravel the mechanisms behind these traditional uses, with studies suggesting that lion’s mane may indeed stimulate nerve regeneration and offer protection against neurodegenerative diseases. Furthermore, its application in supporting digestive health is well documented, as it was traditionally used to treat conditions such as gastritis and ulcers, attributed to its protective effects on the gastric mucosa. The immunomodulatory properties of *Hericium erinaceus* were also acknowledged, as it was believed to bolster the body’s defense mechanisms. Additionally, traditional practitioners valued it for its antifatigue and vitality-enhancing effects, considering it capable of improving energy levels and overall wellbeing. These traditional preparations, including teas, tinctures, and dried powders, have now found echoes in contemporary scientific research, which is progressively validating the historical therapeutic applications of *Hericium erinaceus* [4]. At present, this species is recognized not only for its high nutritional value but also for its diverse biological activities, leading to its widespread use as a functional food and dietary supplement. It is available in various forms, including health wines, beverages, teas, and solid food products, all of which are associated with health benefits such as immune enhancement and improved gastrointestinal digestion [5]. Recent studies have indicated that the bioactive components of *Hericium erinaceus* are being increasingly explored and developed, including diterpenoids [6,7], xanthones, isoflavones [8], and polysaccharides. Among these, polysaccharides are particularly notable due to their high content, extensive applications, and significant industrial potential [8,9,10,11]. Polysaccharides are one of the four fundamental biomolecules essential for life and are found throughout the animal, plant, and microbial kingdoms [12]. HEPs are generally regarded as a primary component in adjunctive therapy for various immune and injury-related diseases, owing to their low toxicity, multifaceted efficacy, and ease of absorption. Furthermore, they offer numerous health benefits and hold promising prospects for applications in food, nutrition, and medicine. In this paper, the research progress of HEPs in terms of extraction, separation, purification, physical and chemical properties, structural characteristics, biological activity, and toxicity are systematically summarized, aiming to fill the gaps in the current research field and provide a solid scientific basis for their further development and application. The safety and efficacy of HEPs are key considerations for their practical application, and it is crucial to ensure their safe use in food and health products. The review of this paper not only helps to promote the wide application of HEPs in related fields but also provides a scientific basis and valuable insights for their utilization in food and industry (Figure 2).

## 2. Extraction, Separation, and Purification

The initial phase in the investigation of polysaccharides involves the process of their extraction, separation, and purification, which is essential for analyzing their physical and chemical properties as well as their pharmacological activities [13,14]. The heterogeneity of HEPs is notable, with variations in their properties primarily attributed to the differing concentrations of ethanol utilized during the purification process. This section provides a comprehensive overview of the pretreatment, extraction, separation, and purification techniques for various types of HEP that have been reported in the recent literature. Detailed information is presented in Table 1.

### 2.1. Extraction

Extraction is an ongoing process that encompasses several stages, including pre-extraction techniques such as drying, crushing, and washing, as well as extraction, which involves both conventional and innovative methods. These processes are designed to enhance the stability of raw materials while simultaneously improving the extraction efficiency and quality of polysaccharides.

Prior to the extraction of polysaccharides from *Hericium erinaceus*, it is essential to conduct standard pretreatment procedures on the specimen. This preliminary step significantly impacts both the quality and yield of the extracted polysaccharides. The process of crushing the material enhances the contact area between the sample and the solvent, thereby facilitating improved extraction efficiency. For instance, comprehensive washing of *Hericium erinaceus* was conducted using deionized water, after which the samples were dried to a constant weight, subsequently crushed, and sieved through a 40-mesh sieve to obtain a uniform powder. This procedure yielded a water-soluble polysaccharide extraction rate of 4.29 ± 0.04% [15]. In a similar manner, *Hericium erinaceus* was subjected to crushing and subsequently passed through a 60-mesh sieve to obtain a uniform powder, resulting in an extraction rate of 5.87 ± 0.16% for the HEP [16]. It is important to acknowledge that the findings presented here are contingent upon the specific experimental conditions and sample sources utilized in these studies. Other variables, such as the drying method, washing duration, and the inherent variability of the mushroom samples, may also exert significant influence on the extraction rate. Therefore, although the results suggest a potential positive correlation between the particle size achieved through crushing and the rate of polysaccharide extraction within a specified range, further research is warranted to fully elucidate this relationship and its underlying mechanisms.

To enhance the purity of crude polysaccharides, it is essential to implement procedures aimed at eliminating small molecular constituents and lipids from the sample prior to the extraction of polysaccharides. A methodology was utilized that involved the pulverization of *Hericium erinaceus*, succeeded by a degreasing procedure employing petroleum ether to extract chromatic compounds and low molecular weight substances [16]. Similarly, Li et al. [17] refluxed the *Hericium erinaceus* powder with ethanol for 8 h prior to HEP extraction to remove monosaccharides and colored substances.

The predominant extraction techniques for HEPs include hot water extraction and alkaline water extraction. The hot water extraction method is widely utilized due to its operational convenience, cost-effectiveness, high efficiency, safety, and low environmental impact. According to Table 1, hot water extraction is frequently employed for HEP extraction, with water temperatures maintained between 70 and 100 °C, extraction durations ranging from 10 min to 36 h, and extraction yields varying from 0.25% to 12.21%. An extraction of HEFB was performed by initially crushing the material, followed by the removal of pigments utilizing 95% ethanol. This was succeeded by an extraction process with distilled water, maintaining a material-to-liquid ratio of 1:15 at a temperature of 95 °C for two separate intervals of three hours each. The yields of HEP-30, HEP-50, and HEP-70, which were precipitated using varying concentrations of ethanol, were measured to be 2.03 ± 0.21%, 1.87 ± 0.11%, and 1.69 ± 0.15%, respectively [18]. Tu’s team [15] extracted *Hericium erinaceus* powder using deionized water at a solid-liquid ratio of 1:20, subsequently performing two extractions at 4 °C for six hours each, and lastly resulting in an extraction rate of HEPs of 4.29 ± 0.04%. These findings underscore the significant impact of the extraction temperature and the ratio of material to liquid on the yield of HEPs. The variables were optimized, revealing that under a material-to-liquid ratio of 1:20, an extraction temperature of 100 °C, and an extraction duration of 1.5 h, the maximum yield of water-soluble polysaccharides achieved was 4.0% [19]. Furthermore, in addition to water, alkaline solutions are commonly utilized for the extraction of HEPs. Alkaline water was employed to extract *β*-glucan from HEFB. Initially, water extraction was performed, after which the fruiting body underwent treatment with a 0.5 M NaOH/0.02% NaBH4 solution (1:20 *w*/*v*) at a temperature of 80 °C for two separate intervals of three hours each. Subsequently, the mixture was neutralized with 36% HCl while being stirred, yielding an extraction efficiency of 7.6% [20]. The HEFB powder was subjected to treatment with a solution of 1.25 M NaOH and 0.05% NaBH4 (at a 1:10 *w*/*v* ratio) at a temperature of 25 °C for two separate intervals of three hours each. This process was subsequently followed by neutralization using 36% acetic acid, resulting in a yield of only 3.1% HEP [21]. These results indicate that the extraction efficiency of the alkaline water method is significantly influenced by factors such as the alkali water concentration, extraction temperature, material-to-liquid ratio, and extraction times.

Water and alkali water extraction represent the predominant methodologies employed for the extraction of HEPs. Nevertheless, the conventional water extraction technique is characterized by its inefficiency, high energy consumption, lengthy processing times, complex procedures, and stringent alkaline extraction conditions, which may compromise the three-dimensional integrity of polysaccharides. Recent advancements have introduced alternative extraction methods, such as microwave-assisted, ultrasonic, and enzyme-assisted techniques, which have addressed some of these limitations to varying degrees.

Microwave-assisted extraction is noted for its rapidity and efficiency, as well as its selective heating capabilities, which contribute to safety and environmental sustainability. This method enhances polysaccharide yields by facilitating the swift and thorough disruption of cell walls. However, it is not without drawbacks, including uneven heating, with the extraction efficiency being contingent upon factors such as the extraction duration, microwave power, duty cycle, and solid-to-liquid ratio. Research conducted by Yu optimized the microwave-assisted extraction process, identifying the optimal conditions for HEP extraction as follows: a temperature of 100 °C, power of 100 W, and an extraction time of 15 min [22].

Ultrasound-assisted enzyme extraction represents a hybrid approach that amalgamates ultrasonic and enzymatic technologies. This method primarily enhances the breakdown of reaction substrates through the cavitation effect induced by ultrasound, coupled with the biocatalytic action of enzymes, thereby facilitating enzymatic catalysis and minimizing waste production. Additionally, the high-frequency vibrations produced by ultrasound increase the collision frequency between enzyme and substrate molecules, thereby accelerating the reaction rate. However, the stability of the enzymes is influenced by variables such as the temperature, pH, and reaction time. Polysaccharides were successfully extracted from *Hericium erinaceus* utilizing an ultrasonic-assisted enzymatic extraction method. Through the application of a Plackett-Burman design and response surface methodology, the researchers identified the optimal parameters for extraction: a duration of 33 min, a liquid-to-material ratio of 30:1, an extraction temperature of 38 °C, a cellulase concentration of 9 g/kg, an ethanol concentration of 20%, and a pH of four. These conditions yielded a polysaccharide extraction rate of 5.87 ± 0.16% [16]. Furthermore, the optimal extraction parameters for soluble dietary fiber from *Hericium erinaceus* were determined through single-factor experiments and a response surface methodology. The findings indicated that a lywallzyme concentration of 1.0%, a compound protease concentration of 1.2%, an ultrasonic duration of 35 min, and ultrasonic power of 150 W resulted in an extraction efficiency of 12.21% [23]. The ultrasound-assisted enzymatic extraction method has gained significant interest due to its simplicity in operation, ease of control, and convenient maintenance of equipment.

### 2.2. Separation and Purification

The biological activity of polysaccharides is significantly influenced by their purity. The preparation of high-purity polysaccharides serves as a critical foundation for investigating the structure and functionality of these compounds, thereby facilitating the development of novel biologically active substances [24]. The initial step in the purification process of polysaccharides involves ethanol fractional precipitation. In a study conducted by Tian et al. [18], the extract of *Hericium erinaceus* was combined, centrifuged, and concentrated, followed by the addition of ethanol to achieve a final concentration of 30%. After allowing the solution to stand, centrifugation, and a series of protein removal procedures, the crude polysaccharide precipitated at this concentration was designated as HEP-30. Subsequently, additional ethanol was introduced to the supernatant to reach a final concentration of 50%, and the purification process was repeated to yield polysaccharide HEP-50. A similar approach was employed to obtain HEP-70 by adjusting the final ethanol concentration to 70%.

In addition to small molecules and pigments, crude plant polysaccharides contained protein impurities. Common techniques for the removal of such contaminants include the Sevag method, trifluoro-trichloroethane method, trichloroacetic acid method, and enzymatic methods, with the Sevag method being most prevalent. Centrifugation was employed to isolate the supernatant from the extract of *Hericium erinaceus*, which was subsequently concentrated through the use of a rotary evaporator, and 95% ethanol solution was introduced at a ratio of 1:4 (*v*:*v*), followed by incubation at 4 °C for 12 h. The resulting precipitate was collected via centrifugation, washed three times with anhydrous ethanol, and treated with Sevag reagent (chloroform: n-butanol, 5:1, *v*/*v*) to remove proteins. The supernatant was subsequently collected, concentrated, and dialyzed at 4 °C using a 1000 Da dialysis membrane. The final solution was concentrated again and freeze-dried to yield HEPs [25]. In another study, the concentrated supernatant was combined with a 10.6% potassium ferrocyanide solution and a 21.9% zinc acetate solution at a ratio of 9:9:100 (potassium ferrocyanide solution, zinc acetate solution, and sample, respectively) to enhance the removal of proteins [26].

Column chromatography is a pivotal technique in the purification of polysaccharides, with ion exchange chromatography and gel filtration chromatography being the most frequently employed methods. Gel filtration chromatography utilizes the molecular sieve effect of porous particles to achieve separation based on the relative molecular mass of the components in the sample, employing materials such as dextran and agarose gel columns. Ion exchange chromatography, on the other hand, is a method for the separation and analysis of cations and anions in the solution, integrating ion exchange principles with liquid chromatography technology. Anion exchange chromatography is particularly effective for separating neutral and acidic polysaccharides, utilizing columns such as DEAE-agarose or DEAE-cellulose. The centrifugal extract was subjected to hollow fiber ultrafiltration to remove small molecules and neutralize the excess sodium chloride. The solution was then vacuum-concentrated at 60 °C and freeze-dried for 12 h to obtain alkali-soluble HEP. This alkali-soluble HEP was dissolved in distilled water and subjected to elution through a DEAE-cellulose column to isolate a neutral polysaccharide fraction and an acidic polysaccharide fraction (AHEP-A) using 0.3 M NaCl. AHEP-A underwent further purification via molecular exclusion chromatography using an agarose gel column (CL-6B, 2.6 cm × 100 cm) eluted with 0.15 M NaCl at a flow rate of 0.4 mL/min to yield a homogeneous fraction (AHEP-A-b) [20]. Furthermore, the water-soluble total polysaccharide of *Hericium erinaceus* was dissolved in distilled water, subsequently subjected to separation via a DEAE-cellulose column, and eluted with distilled water to isolate the neutral polysaccharide. This was further purified using a Sepharose CL-6B column, and the primary product underwent additional purification via a Sephadex G-75 column to isolate the purified polysaccharide [27]. The crude polysaccharide was applied to a DEAE Sepharose Fast Flow column, utilizing a mobile phase consisting of distilled water and 1 M NaCl in a stepwise gradient. This process resulted in the isolation of four distinct fractions, designated as HEP-1, HEP-2, HEP-3, and HEP-4 [28].

**Table 1:** Pretreatment of different kinds of *Hericium erinaceus*, extraction, and separation and purification methods of polysaccharides.

Type	Pretreatment		Polysaccharide Names	Extraction Method					Separation and Purification Methods	Reference
	Drying Condition	Granularity (Item)		Solid/Liquid Ratio (*w*:*v*)	Extraction Frequency	Total Extraction Time	Extraction Temperature (°C)	Extraction Rate		
Dried *Hericium erinaceus* powder	-	-	HEP	1:10	1	6 h	100	-	95% ethanol precipitation Sevag method for protein precipitation	[25]
HEFB	40 °C, 6 h	24	HEP	-	1	4 h	80	-	Ethanol precipitation Sevag method for protein precipitation DEAE-52 column Sephadex G-100 gel permeation column	[17]
Dried HEFB	-	80	HEFPs	1:40	1	4 h	80	-	Precipitate proteins with 10.6% potassium ferrocyanide and 21.9% zinc acetate solution at a 9:9:100 ratio (potassium ferrocyanide, zinc acetate, and sample, respectively), then dialyze with distilled water Ethanol precipitation	[26]
HEM	40 °C	-	PFHE	1:10	1	24 h	70	-	Lipid removal with 96% ethanol	[29]
HEFB	-	-	Water-soluble polysaccharides	1:15	1	2 h	100	-	Ethanol precipitation	[30]
HEFB powder after semi-solid enzymolysis	80 °C	-						-	-	
Dried and ground *Hericium erinaceus*	-	-	HEP-W	1:15	1	2 h	100	0.3%	Sevage method for protein precipitation Ethanol precipitation	[21]
	-	-	HEP-A	1:10	2	6 h	25	3.1%	Acid precipitation Sevage method for protein precipitation Ethanol precipitation	[21]
*Hericium erinaceus*	-	100	*Hericium erinaceus* polysaccharide	3:100	1	3.5 h	50	-	-	[31,32]
HEFB	50 °C, 10 h	50	HEP	1:15	2	6 h	95	-	Ethanol precipitation Sevag method for protein precipitation	[33]
HEFB	50 °C, 10 h	50	HEP50	1:15	2	6 h	95	1.87 ± 0.11%		[34]
HEFB	Freeze-dried	-	*Hericium erinaceus* polysaccharide	1:1	1	10 min	100	-	-	[14]
Dried HEFB	-	-	*Hericium erinaceus* polysaccharide	1:30	4	4 h	100	-	-	[35]
HEFB	-	-	HEP-1, HEP-2, HEP-3, HEP-4, HEP-5	1:10	1	8 h	100	2.735%	Ethanol precipitation DEAE Cellulose-52 column Sephadex G-100 column	[36]
Fermented HEM	-	-	PHEB	-	2	6 h	80	-	Ethanol precipitation Sevag method for protein precipitation DEAE Sepharose Fast Flow HiLoad 16/600 Superdex 200 prep grade Column	[32,37]
Dried HEFB	-	-	HEPs	1:10	1	8 h	100	2.735%	-	[38]
HEM	-	-	wHEP-1, wHEP-2, wHEP-3	1:5	3	12 h	80	-	Ethanol precipitation 3-K hollow fiber ultrafiltration cartridge DEAE-Sephadex A-50 column 0.2-K hollow fiber ultrafiltration column P30 column	[39]
*Hericium erinaceus* residue	-	-	HRPs	3:50	1	4 h	90	-	Ethanol precipitation Sevage method for protein precipitation	[40]
HEFB	-	-	HEP 1, HEP 2, HEP 3, HEP 4	1:20	2	4 h	100	5%	95% ethanol precipitation Sevag method for protein precipitation DEAE Sepharose Fast Flow column	[28]
HEFB	50 °C, 72 h	100	HEFP-2b	1:1	1	3 h	80	-	Ethanol precipitation DEAE-cellulose-52ion exchange column A gel permeation chromatography column of Sephacryl S-400	[41]
HEFB	50 °C, 72 h	100	HEFPs	1:40	1	3 h	80	-	10.6% potassium ferricyanide Solution, 21.9% zinc acetate solution, and the concentrated supernatant were mixed at a 1:1:10 ratio, precipitation Ethanol precipitation	[42]
Dried *Hericium erinaceus*	-	-	HEP	1:20	2	4 h	110	-	Ethanol precipitation Sevage method for protein precipitation anion-exchange chromatography Sephadex G-100 column	[43]
Cultured HEM	-	-	wHEP-1	1:5	3	36 h	80	-	Ethanol precipitation A hollow fiber ultra-filtration cartridge 0.2 K hollow fiber ultrafiltration column DEAE Sephadex A-50	[44]
The mature HEFB	-	-	H6PC20	1:2	2	4 h	100	0.25%	Ethanol precipitation	[45]
Cultured HEM	-	-	Hep-1, Hep-2, Hep-3	-	2	12	70	-	Ethanol precipitation Hollow-fiber ultrafiltration cartridge DEAE-Sephadex A-50 column Bio-Gel P-30	[46]
HEM	-	-	EP-1	1:5	3	12	70	-	Ethanol precipitation hollow fiber ultrafiltration cartridges (3 K) hollow fiber ultrafiltration column (0.2 K) DEAE-Sephadex column	[47]
Cultured HEM	-	-	EP-1	1:5	3	12	70	-	Ethanol precipitation Hollow fiber ultrafiltration cartridges (3 K) Hollow fiber ultra-filtration column of 0.2 K DEAE-Sephadex column	[48]

“-” indicates that information is not available in the literature.

## 3. Physicochemical Properties and Structural Characteristics

Polysaccharides are high molecular weight compounds that are synthesized from numerous monosaccharide units linked by glycosidic bonds. The physicochemical properties and structural attributes of polysaccharides are primarily determined by factors such as the monosaccharide composition, molecular weight, conformational characteristics, and chemical structures, including the type and configuration of glycosidic bonds. A summary of the physicochemical properties and structural characteristics of HEPs in recent years is presented in Table 2.

### 3.1. Monosaccharide Composition

The types of monosaccharides serve as a fundamental basis for the structural characteristics of polysaccharides, which are intrinsically linked to their biological activities. Prior to analyzing the monosaccharide composition, it is necessary to hydrolyze the sample to ensure the complete cleavage of glycosidic bonds within the polysaccharide. Various hydrolysis methods are employed, including methanol hydrolysis, hydrochloric acid hydrolysis, sulfuric acid hydrolysis, and trifluoroacetic acid hydrolysis. Following hydrolysis, the polysaccharide undergoes a series of treatments, including neutralization, filtration, and derivatization. The polysaccharides Hep-1 and Hep-2, which are extracted from HEM, are composed of glucose and galactose. In contrast, Hep-3, characterized as dextran, is composed exclusively of glucose [46]. Currently, determination of a monosaccharide’s composition is predominantly performed using gas chromatography (GC), gas chromatography-mass spectrometry (GC-MS), or high-performance liquid chromatography (HPLC). For instance, gas chromatography was employed to examine the enzymatic hydrolysate of HEP (EHEP), which demonstrated that its primary constituents were glucose, rhamnose, mannose, and galactose [49]. Utilizing 2M trifluoroacetic acid (TFA), the hydrolysis of HEPs into monosaccharides was conducted at a temperature of 121 °C for a duration of six hours. This was followed by derivatization using acetaldehyde nitrile acetate, after which GC-MS analysis was performed. Their findings indicated that the polysaccharides from HEFB were predominantly composed of fucose, galactose, and glucose [36]. Additionally, a study was undertaken in which the hydrolysate was subjected to repeated evaporation to dryness using methanol following the hydrolysis of the polysaccharide fraction with an acidic aqueous solution, aimed at removing any residual acid. The resulting monosaccharide mixture was converted into sugar alcohol acetates and analyzed using a Varian 450-GC chromatograph equipped with a flame ionization detector. Their comparative analysis revealed that regardless of the substrate used for cultivation, the monosaccharide composition of the polysaccharide fraction from *Hericium erinaceus* consistently included arabinose and glucose [29]. In comparison with traditional methodologies, HPLC utilizing a C18 chromatographic column was employed to analyze the water-soluble oligosaccharides derived from *Hericium erinaceus* (wHEP-1). This analysis revealed the monosaccharide composition to consist of glucose, mannose, and galactose [44]. High-performance anion exchange chromatography and size exclusion chromatography were employed to analyze the monosaccharide composition of polysaccharides derived from both HEFB and its enzymatic hydrolysate subsequent to semi-solid enzymatic hydrolysis. Their results indicated that both the water-soluble polysaccharides from the fruiting body and the enzymatic hydrolysate contained fucose, galactose, glucose, and xylose, with the latter exhibiting a unique composition that included glucosamine [30]. As summarized in Table 2, it has been reported that HEP predominantly contains galactose and glucose, with potential occurrences of mannose and fucose, while fructose, glucuronic acid, glucosamine, glucosamine hydrochloride, xylose, and arabinose are present in extremely limited quantities. Furthermore, studies have established correlations between monosaccharide composition and pharmacological activities. Notably, glucose has been associated with enhanced cell protection, improved cell viability, and stronger gastric protective effects [50]. Conversely, a significant negative correlation was observed between the total antioxidant capacity and the presence of fucose and galactose, suggesting that these monosaccharides collectively influence the total antioxidant capacity [51]. This relationship elucidates the pharmacological activities of polysaccharides from the perspective of their monosaccharide compositions.

### 3.2. Molecular Weight

The molecular weight serves as a critical parameter for the characterization of polysaccharide structures, as illustrated in Table 2. Typically, the molecular weight of polysaccharides is assessed using high-performance gel permeation chromatography (HPGPC), which may also be referred to as high-performance size exclusion chromatography. This analytical technique is generally equipped with various detectors, including a refractive index detector (RI), a multi-angle light scattering detector (MALS), or an ultraviolet detector (UV), to facilitate precise analysis of the molecular weight distribution of polysaccharides. Notably, the MALS can yield absolute molecular weight values independent of standard reference samples. Research conducted by Qiao established that the AHEP-A-b was 20 kDa, as determined by HPGPC [51]. A weight-average molecular weight of 16.7 kDa was reported for a low molecular weight polysaccharide derived from HEFB, employing high-performance liquid gel permeation chromatography coupled with a differential refractive index detector [52].

In recent years, researchers have increasingly employed combinations of various chromatographic columns and detectors to measure polysaccharide molecular weights based on specific requirements. For instance, a gel chromatography-differential refractometer-multi-angle laser light scattering system was employed to determine that the molecular weight of the HEP was 23.5 kDa, with an average particle size of approximately 408 nm [16]. Furthermore, a gel exclusion chromatography column was integrated with differential and laser scattering detectors to assess the homogeneity and molecular weights of polysaccharides obtained from the fermentation HEM (PHEB), yielding an average molar mass of 36.1 kDa [37]. Additionally, a size exclusion chromatography column was combined with a multi-angle light scattering detector and a refractive index detector to analyze the weight-average molecular weight of water-soluble *β*-glucan [28]. Lastly, three gel permeation columns were connected in series, which facilitated the determination of the molecular weight of the HEP to be 43 kDa using a differential refractive index detector [43].

### 3.3. Conformational Characteristics

SEM is presently the predominant technique employed for examination of the conformational properties of polysaccharides, owing to its high resolution and magnification capabilities. This method facilitates the acquisition of detailed images of the sample surface by scanning the signals produced from the interaction between the electron beam and the sample, thereby enabling observation of the surface morphology and microstructure. The resolution achieved can reach the nanometer scale, as detailed in Table 2. Research conducted by the findings indicated that the HEP displayed a stratified architecture characterized by limited fragmentation, a loosely arranged configuration, and a polished surface. This suggests significant interaction among polysaccharide molecules, which led to the formation of aggregates [15]. Employing SEM, the study examined the aggregates of *Hericium erinaceus* polysaccharide (HEPN), which exhibited a pronounced luster and an irregular, heterogeneous distribution of particles [53]. Furthermore, the SEM imagery of polysaccharides derived from HEFB (HEFPs) revealed a rough and massive surface with a dense honeycomb structure at a magnification of 2000×. SEM also provides three-dimensional representations of the sample surface, facilitating the identification and analysis of the sample [42]. A comparative analysis was performed on the microstructures of three polysaccharide components (HEP-30, HEP-50, and HEP-70) derived from *Hericium erinaceus*. Their SEM observations indicated that the surface morphology of these polysaccharides was predominantly flat, layered, smooth, and wrinkled, with significant variations in shape and size among the three components. Notably, the polysaccharide structure of HEP-30 was found to be slightly larger and sparser than the other two, with HEP-50 following closely, a finding that may be attributed to differences in molecular weight [18]. In contrast to SEM, AFM generates images through the interaction between a probe and the sample surface, making it suitable for examining the surface morphology and mechanical properties while providing authentic three-dimensional surface images. The sample preparation requirements for AFM are relatively straightforward and do not necessitate complex pretreatment. Specific details are presented in Table 2. The surface morphology of EHEP was analyzed utilizing AFM, revealing the presence of conical aggregates [49]. The integration of both SEM and AFM techniques can yield a more comprehensive understanding of the conformational characteristics of the sample. The surface morphology of the heteropolysaccharide fraction derived from *Hericium erinaceus* (HEP-W) was characterized utilizing SEM under high-vacuum conditions, with the acceleration voltage set to 10 kV. This analysis unveiled a prominent fibrous network structure. AFM imaging was conducted in tapping mode, demonstrating that the HEP-W exhibited a branched, flexible random coil conformation [54]. Additionally, it was reported that the SEM analysis demonstrated a smooth and transparent surface for HEPs, whereas AFM imaging illustrated an irregular surface topography, which may include multiple sharp edges and uneven protrusions [43].

### 3.4. Chemical Structure Analysis

In addition to the composition of monosaccharides, the molecular weight, conformational characteristics, and chemical structure are critical structural attributes of polysaccharides. The fundamental chemical structure of an HEP can be elucidated through various analytical techniques, including nuclear magnetic resonance (NMR) spectroscopy, electrospray ionization tandem mass spectrometry (ESI-MS/MS), GC-MS, and Fourier transform infrared spectroscopy (FT-IR). Detailed findings are presented in Table 2. FT-IR is the most prevalent method for analyzing polysaccharide structures, as it provides comprehensive insights into the chemical structure, including functional group identification and glycosidic bond types. Characteristic absorption peaks corresponding to O-H stretching vibrations and C-H asymmetric and symmetric stretching vibrations are evident in the IR spectra of polysaccharides, serving as a fundamental basis for structural identification. The FT-IR spectrum of HEP10 revealed three distinct absorption peaks at 1137.9 cm^−1^, 1076.2 cm^−1^, and 1024.1 cm^−1^, which suggest the presence of pyranose and furanose structures. A minor absorption peak near 890 cm^−1^ was associated with the *β* configuration of the glucose unit, while the peak at 842.8 cm^−1^ signified the presence of an *α*-glycosidic bond [55]. An FT-IR analysis was performed on three principal polysaccharides (wHEP-1, wHEP-2, and wHEP-3) extracted from HEM, which demonstrated a shared broad peak, suggesting the presence of analogous functional groups among the polysaccharides. Absorption peaks at 858 and 777 cm^−1^ confirmed the presence of *β*-glycosidic and *α*-glycosidic bonds, respectively [39]. The infrared spectra of HEM polysaccharide (HMP) and HEFB polysaccharide (HFP) demonstrated that both the HMP and HFP exhibited characteristic polysaccharide structures, with strong absorption peaks near 1022 cm^−1^. Both structures were identified as pyran ring configurations [56]. Additionally, FT-IR was employed to analyze the chemical bonds and functional groups present in the HEPs and their derivatives. The findings indicated that the polysaccharides extracted from *Hericium erinaceus* through both water and alkali methods were identified as pyran ring polysaccharides featuring *β*-glycosidic bonds, which exhibited analogous functional groups. The absorption peaks observed at 1023.32 cm^−1^ and 1200.22 cm^−1^ provided evidence for the existence of *α*-pyran rings, while the peaks at 892.56 cm^−1^ and 923.45 cm^−1^ confirmed the presence of *β*-glycosidic bonds [21].

GC-MS has also been extensively employed for the identification of polysaccharides. The *β*-glucan obtained from HEFB was subjected to methylation, hydrolysis, and acetylation, followed by an analysis using GC-MS. This analysis revealed that the main chain of the alkaline *β*-glucan was composed of (1→6)-glucan, with terminal glucose residues and (1→3)-linked glucose (8.2%) potentially connected to the side chain [20]. Moreover, GC-MS is advantageous for analyzing different fractions of the same crude polysaccharide. Utilizing GC-MS, the study examined various HEPs and determined that HEP fraction-1 (HEP-1) was primarily composed of O-3-branched (1→6)-*β*-D-glucopyranose. Additionally, it contained minor quantities of *α*-D-glucopyranose, *β*-D-glucofuranoses, and *α*-L-fucose, which were associated with the oxygen atom at the C-2 position of the (1→6)-*α*-D-galactopyranose main chain. Similarly, HEP-2 and HEP fraction-3 (HEP-3) were characterized by (1→6)-linked-*α*-D-Galp backbones and (1→6)-linked-*β*-D-Glcp branches, although their residues differed. HEP-2 was composed of →1)-Fruf-(2→1)-*α*-D-Glcp, while HEP-3 consisted of →1)-*β*-D-Galp-(6→1)-*α*-D-Glcp. Additionally, the main chain of HEP-4 was identified as comprising (1→6)-linked furan galactose residues, which were substituted by (1→6)-linked *α*-D-glucopyranose side chains at C-3. HEP-5 contained a (1→4)-linked-*α*-Glc and an *α*-D-glucopyranose residue. In comparison with GC-MS, ESI-MS/MS is capable of detecting substances at microgram or lower concentrations, making it more suitable for trace analysis [36]. Employing ESI-MS/MS, the study determined that the structural composition of HEP-2 primarily consists of 2–7 hexose oligosaccharides, with the pentasaccharide of HEP-2 characterized by a 1→6 linkage of the Gal type [46].

NMR is a widely utilized analytical technique that provides information regarding the number and type of chemical functional groups within molecules, as well as the connectivity between atoms, thereby aiding in the determination of molecular structures. HEP-1 was isolated from HEFB, and subsequent NMR analysis was performed. The 13C-NMR spectrum exhibited a signal at 100–103 ppm, indicating the presence of a *β*-glycosidic bond, while the absence of a signal in the 90–100 ppm range suggested the lack of an *α*-glycosidic bond. It was inferred that the structure of HEP-1 is →6)-*β*-D-Glcp-(1→), with the side chain primarily connected to →3,6)-*β*-D-Glcp-(1→) and predominantly linked to *β*-D-Glcp-(1→) [52]. Through NMR analysis of wHEP-1, an oligosaccharide that is soluble in water and extracted from *Hericium erinaceus*, the spectrum revealed four heteroproton signals at δ 5.30, 5.11, 4.84, and 4.53 ppm. The chemical shifts at 5.30 and 5.11 ppm were attributed to *α*-glycosidic bonds, while the signals at δ4.84 and 4.53 ppm were designated as *β*-glycosidic bonds. These findings corroborate the FT-IR spectra, further indicating that wHEP-1 possesses both *α* and *β* configurations [44].

**Table 2:** Physicochemical properties and structural characteristics of *Hericium erinaceus* polysaccharides.

Polysaccharide Name	Monosaccharide Composition	Mw (kDa)	Conformational Characteristics		Structural Analysis Techniques and Results			Reference
			SEM	AFM	FT-IR	MS	NMR	
HEP	Rha, Fru, Gal, Glc	23.5	-	-	O-H stretching vibration, C-H asymmetric stretching vibration, bound water, O-H bending vibration of carboxyl group, C-O glycosidic bond, stretching vibration of pyran ring, *α*-glycosidic bond and *β*-glycosidic bond, C-C stretching vibration.	-	-	[16]
AHEP-A-b	Glc, Gal, Man, GlcA, Fuc	20	-	-	O-H stretching vibration, C-H stretching vibration, bound water and C = O groups, variable angle vibration of C-H groups, pyranose ring, *β*-linked glycosyl residues.	The polymerization degree of the [→3)-*β*-D-Glcp-(1→)] side chain is 2~8.	The main chain is composed of [→6)-*β*-D-Glcp-(1→], and the side chain is composed of [→3)-*β*-D-Glcp-(1→] and *β*-D-Glcp-(1→] linked to the C-3 main chain of Glcp.	[20]
HEP	Glc, Man, Gal, GlcN	27.5	The dense and smooth surface has a layered structure, which is characterized by small and rough fragments.	-	O-H stretching vibration, C-H stretching vibration, C-O-C stretching vibration of pyranose, C-O-H or C-O-C bending vibration of C-O bond.	-	-	[25]
HEP	Man, Rha, Glc, Gal, Fuc	-	-	-	O-H antisymmetric stretching vibration, C-H symmetric stretching vibration, carbonyl (C = O) antisymmetric stretching vibration, C-H antisymmetric bending vibration, pyranose ring.	-	-	[17]
HEFPs	Fuc, GlcN·HCl, Gal, Glc, Xyl, Man	17.21	-	-	O-H stretching vibration, C-H stretching vibration, COO-asymmetric stretching vibration, pyranose ring.	-	-	[26]
HEP10	Fuc, Ara, Gal, Glc, Man, Xyl	9.9	-	-	O-H stretching vibration, C-H stretching vibration, asymmetric and symmetric stretching vibration of carboxyl group, pyranose ring, furanose ring, *β* configuration, *α*-glycosidic bond.	-	The glycosidic linkage of HEP10 might be (1→2) and (1→6).	[55]
Gal-HE	Fuc, Gal, Glc, Man	22.1	-	-	-	-	Mannan galactomannan is composed of a 1,6-linked *α*-D-Gal backbone, which contains a small amount of *α*-1,6-D-Me-Galp, and is branched by a single-unit *β*-Manp residue at O-2. Fucoidan contains *α*-1,6-linked DGal, and the t-*α*-L-Fucp side chain is replaced at O-2.	[21]
HMP	-	-	Granular structure, with rough surface and irregular crystal morphology.	-	O-H stretching vibration, C-H stretching vibration, *β*-pyran bond, pyranose ring appeared.	-	-	[56]
HFP	-	-	Block structure, smooth surface, regular crystal morphology.	-		-	-	
HEP-30	Fru, Man, Glc, Gal	15.019 ± 1.59	The surface morphology of the three polysaccharides was mainly flat, layered, smooth, and wrinkled. The polysaccharide structure of HEP-30 was slightly larger and sparser than those of the other two polysaccharides.	-	O-H stretching vibration, C-H stretching vibration, C = O group or C = C group, C-H variable angle vibration, pyranose ring appeared.	-	-	[18]
HEP-50	Fru, Man, Glc, Gal	16.723 ± 2.11		-		-	-	
HEP-70	Fru, Man, Glc, Gal	4.771 ± 0.21		-		-	-	
PHEB	Gal, Glc, Man, GlcA	36.1	-	-	O-H stretching vibration, C-H stretching vibration, crystal water, C-O symmetric stretching vibration, O-H variable angle vibration, pyranose ring.	-	There exists →6)-*α*-D-Galp-(1→6)-*α*-D-Galp-(1→).	[37]
HEP	Man, Gal, Glc, Fuc, GlcA	19.7	Layered, with a small amount of debris, loose shape, and a smooth surface.	-	O-H stretching vibration, C-H stretching vibration, − C = O stretching vibration, pyranose ring, *β*-pyranoside bond, *α*-glycosidic bond.	-	Gal, Fuc.	[15]
HEP	Xyl, Ara, Fuc, GalA, Man, Glc, Gal	-	-	-	-	-	-	[22]
wHEP-1	Man, Glc, Gal	5.01	-	-	O-H stretching vibration, C-H stretching vibration, *β*-glycosidic bond, *α*-glycosidic bond.	-	*α*-glycosidic bond, *β*-glycosidic bond.	[39]
wHEP-2	Glc, Gal	1.812	-	-		-		
wHEP-3	Glc, Gal	1.11	-	-		-		
SHRPs	Xyl, Ara, Rha, Fuc, Man, Gal, Glc, GlcA	-	-	-	O-H stretching vibration, C-H stretching vibration, C = O bond in carboxyl group, stretching vibration of uronic acid, furan sugar ring, *β*-glycosidic bond.	-	-	[40]
HEP-1	Fuc, Gal, Glc	-	-	-	-	-	-	[28]
HEP-2	Fuc, Gal, Glc	-	-	-	-	-	-	
HEP-3	Glc	13.3	-	-	-	-	The *β*-glucan could be tentatively deduced as a (1→6)-linked glucan main chain with (1→3)-linked glucopyranosyl branching unit(s).	
HEFP-2b	Fuc, Gal, Glc, Man	32.52	Flat and smooth shape, and the particles are layered.	-	O-H stretching vibration, C-H stretching vibration, C-H bond deformation vibration, C = O stretching vibration, pyranose ring, *β* and *α* configurations.	-	The backbone of HEFP-2b might mainly consist of (1→6)-linked-*α*-D-glucose residues, (1→4)-linked-*β*-D-galactose residues, (1→3,6)-linked-*α*-D-mannose residues, and terminal glucose and fucose. Two branches consisted of (1→6)-linked-*β*-D-galactose and (1→3)-linked-*α*-D-mannose residues.	[41]
HER	-	-	Aggregate and irregular shape, rough surface.	-	-	-	-	[57]
HEFPs	Ara, Gal, Glc, Man	-	Rough and blocky surface with dense honeycomb structure.	-	O-H stretching vibration, C-H stretching vibration, C = O stretching vibration, C-H bond deformation vibration, pyranose ring.	-	-	[42]
HEP_N_	Man, Glc, Gal	12.713	Bright luster, showing irregular, uneven distribution of particles, concave, wrinkled, and relatively fluffy.	-	O-H stretching vibration, C-H stretching vibration, bound water stretching vibration, C = O stretching vibration, in-plane bending vibration of C-H, glycosidic bond and *α* configuration.	-	*β*-D-Glc and *α*-D-Gal.	[53]
H6PC20	Glc	-	-	-	-	-	H6PC20 has a backbone of (1→3)- linked-*β*-D-glucopyranosyl units, with one single-unit *β*-D-glucopyranosyl branch substituted at O-6 on every third backbone unit.	[45]
EP-1	-	3.1	-	-	-	-	-	[47]

Mw = molecular weight, Glc = glcose, Gal = galactose, Man = mannose, Xyl = xylose, Rha = rhamnose, GlcN = glucosamine, GlcA = glucuronic acid, Fuc = fucose, Ara = arabinose, GalA = galacturonic acid, and Fru = fructose. “-” indicates that information is not available in the literature.

## 4. Biological Activity

**Figure 3:**
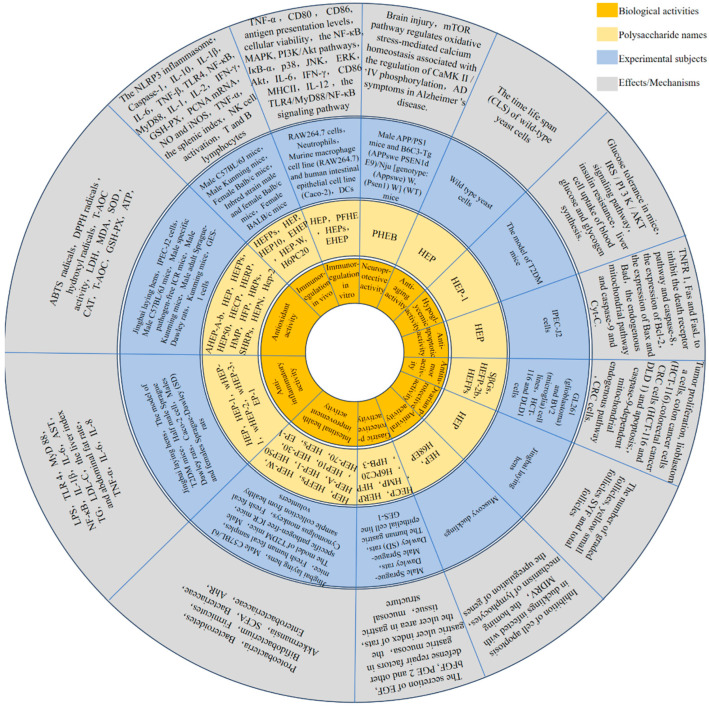
Schematic diagram of HEPs’ biological activity, experimental subjects, and mechanisms of action, showing differentiation 86 (CD86), differentiation 80 (CD80), major histocompatibility complex class II (MHCII), interleukin-12 (IL-12), nuclear factor kappa-light-chain-enhancer of activated B cells (NF-κB), mitogen-activated protein kinases (MAPKs), c-Jun N-terminal kinase (JNK), extracellular signal-regulated kinases (ERKs), protein kinase B (PKB), interleukin-6 (IL-6), interferon-γ (IFN-γ), cell counting kit-8 (CCK-8), cyclophosphamide (Cy), interleukin-1 beta (IL-1β), nitric oxide (NO), inducible nitric oxide synthase (iNOS), lipopolysaccharide (LPS), 2,2′-Azino-bis (3-ethylbenzothiazoline-6-sulfonic acid) (ABTS), 2,2-Diphenyl-1-picrylhydrazyl (DPPH), oxidative-sensitive dye 2′,7′-Dichlorofluorescin Diacetate (DCFH-DA), the total antioxidant capacity (T-AOC), malondialdehyde (MDA), glutathione peroxidase (GSH-PX), superoxide dismutase (SOD), catalase (CAT), adenosine triphosphate (ATP), high-fat diets (HFDs), toll-like receptor 4 (TLR4), aspartate aminotransferase (AST), triglycerides (TGs), low-density lipoprotein cholesterol (LDL-C), short-chain fatty acids (SCFAs), aryl hydrocarbon receptor (AhR), interleukin-22 (IL-22), epidermal growth factor (EGF), high-sugar diet (HFSD), nerve growth factor (NGF), Muscovy duck reovirus (MDRV), yellow small follicles (SYFs), luteinizing hormone (LH), estradiol (E2), and follicle-stimulating hormone (FSH).

*Hericium erinaceus*, commonly known as lion’s mane mushroom, is a valuable edible fungus. One of its significant bioactive components is HEPs. Recent research has demonstrated that HEPs exhibit a range of beneficial properties, including immunomodulatory, anti-tumor, anti-inflammatory, antioxidant, gastrointestinal health enhancement, gastric protection, neuroprotective, and antiviral effects, as shown in Figure 3 (Table 3).

### 4.1. Immunomodulatory Activity

**Figure 4:**
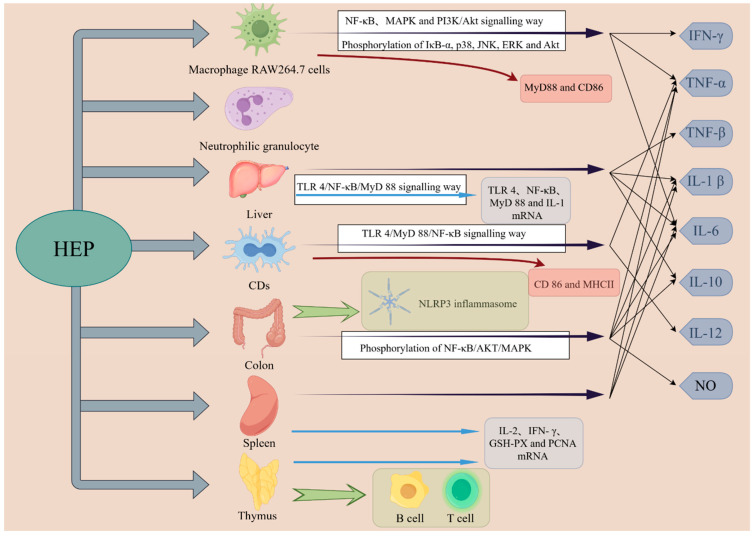
Schematic diagram of HEPs’ immune activity mechanism potential.

The thymus and spleen serve as critical central and peripheral immune organs in animals, playing essential roles in mediating both cellular and humoral immunity. The differentiation and maturation of T lymphocytes take place within the thymus [59]. Cytokines, which are immune regulatory factors predominantly produced by T lymphocytes, facilitate intercellular communication and modulate immune responses and cellular activities. These cytokines interact within intricate networks to sustain immune homeostasis and are vital for the diagnosis and treatment of human health conditions [60]. A schematic representation of the immune activity of HEPs is illustrated in Figure 4.

#### 4.1.1. Immunoregulation In Vitro

In vitro immunological studies are essential for understanding immune function. Research conducted by Yu demonstrated that EHEP enhances the expression of CD86 and MHCII in DCs, as well as increasing the levels of IL-12 and TNF-α. This process further promotes the morphological changes and immune activity of DCs by activating the TLR4/MyD88/NF-κB signaling pathway [61]. The immunomodulatory effects of HEP-1 activate the NF-κB, MAPK, and PI3K/Akt pathways, leading to the phosphorylation of IκB-α, p38, JNK, ERK, and PKB, which in turn results in the production of TNF-α, IL-6, and IFN-γ, thereby indicating the significant immunomodulatory potential of HEP-1 [36]. Additionally, polysaccharide fractions derived from the fungus *Hericium erinaceus*, cultivated on various grain matrices, could enhance the phagocytic and bactericidal activities of neutrophils [29]. Furthermore, Shi et al. [16] employed the CCK-8 assay to assess the viability of the macrophage cell line RAW264.7 following treatment with HEPs. The findings revealed that HEPs significantly stimulated the proliferation of RAW264.7 cells within the concentration range of 25–100 μg/mL. Concurrently, the study indicated a marked increase in the phagocytic activity of macrophages treated with HEPs, along with elevated levels of TNF-α and IL-6 cytokines. Additionally, there was a significant upregulation of the cluster of CD80 and CD86 on the surface of the macrophages, as well as an increase in antigen levels, which collectively enhanced the potential to elicit an immune response.

#### 4.1.2. Immunoregulation In Vivo

Immune organs and cytokines are critical components of the immune response. HEPs have been shown to significantly influence immune organs and cells, particularly the spleen. Research indicates that the thymic microstructure in mice treated with HEPs exhibited notable improvements [62]. Furthermore, studies demonstrated that both HEPs and their enzymatically hydrolyzed form can enhance the activation of peritoneal macrophages in mice, thereby initiating an immune response, with the enzymatic hydrolysis of HEPs displaying a more pronounced effect [49]. Investigations by Wu et al. [54] revealed that the heteropolysaccharide fraction of *Hericium erinaceus* not only increased the spleen index in mice subjected to Cy but also upregulated the activation of natural killer (NK) cells and promoted the proliferation and activation of T and B lymphocytes in immunosuppressed mice, as well as enhancing the phagocytic activity of peritoneal macrophages, thus improving overall immunity. Additionally, β-glucan (H6PC20) not only stimulated the proliferation of lymphocytes in mouse spleens but also significantly elevated the expression of inflammatory cytokines such as TNF-α, IL-6, and IL-1β, suggesting that H6PC20 possesses substantial immune-enhancing properties and may serve as a potential health food resource [45]. In addition, it was further established that HEP treatment can significantly increase liver indices, facilitate the repair of liver damage to some extent, and effectively promote the secretion of inflammatory factors (serum IL-1β, IL-6, IL-10, and TNF-β) based on animal experimental data. Immune factors act as communicative messengers among immune system cells, regulating and coordinating immune cell activity and enhancing overall immunity [33].

HEPs also play a significant role in the context of ulcerative colitis. Animal experiments have been conducted that demonstrated the efficacy of HEFPs in alleviating ulcerative colitis in murine models. This effect is achieved through the inhibition of NLRP3 inflammasome formation and the downregulation of caspase-1 expression, both of which are critical components of the innate immune system. Concurrently, ELISA results indicated that HEFPs downregulate pro-inflammatory cytokines while upregulating the anti-inflammatory cytokine IL-10, thereby playing a crucial role in modulating the immune response in vivo and suggesting its potential to restore immune balance in ulcerative colitis [26]. Low molecular weight HEPs can inhibit the production of NO, iNOS, and inflammatory cytokines (including TNF-α, IL-1β, and IL-6) stimulated by LPS and dextran sulfate sodium (DSS) as well as inhibit activation of the NLRP3 inflammasome and phosphorylation of NF-κB, AKT, and MAPK, thereby enhancing immune function and indicating its potential therapeutic efficacy for acute ulcerative colitis [55].

### 4.2. Antitumor Activity

Chemotherapy is a prevalent therapeutic approach for cancer that targets and eliminates malignant cells through pharmacological agents that inhibit their proliferation and dissemination. However, these chemotherapeutic agents also adversely affect normal cells, resulting in a range of side effects, including nausea, vomiting, alopecia, and immunosuppression. Certain natural products exhibit promising anti-tumor properties with minimal adverse effects [63]. For instance, research conducted by Liu demonstrated that the HEP fraction (HEFP-2b) significantly inhibits the proliferation of colon cancer cells. Cell cycle arrest experiments indicated that the primary mechanism of HEFP-2b involves inducing cell cycle arrest in the S phase of HCT-116 colon cancer cells [41]. Furthermore, it was reported that HEFPs markedly increase the levels of reactive oxygen species in colorectal cancer cells (HCT-116 and DLD-1), leading to apoptosis via a caspase-9-dependent mitochondrial intrinsic pathway and thereby providing an effective treatment modality for colorectal cancer. This research offers critical insights for future investigations into HEPs as a potential functional food component or pharmaceutical agent for colon cancer therapy [42]. Additionally, other studies have indicated that the gut microbiota can modulate the efficacy and toxicity of chemotherapy and immunotherapy agents, suggesting that the intestinal microbiome may play a supportive role in enhancing anti-tumor effects and facilitating comprehensive, multi-faceted therapeutic strategies for cancer treatment [64].

In the context of brain cancer, HEPs also demonstrate distinct advantages. The brain possesses a specialized immune system, and research has indicated that effective glioblastoma treatment may involve inhibition of the immune microenvironment. Glioma-associated microglia and infiltrating macrophages (GAMs), which constitute a significant portion of the tumor mass, are critical targets for immunotherapy [65,66]. In this regard, *β*-glucans isolated from a range of fungal sources, such as *Hericium erinaceus*, have been shown to inhibit tumor proliferation and promote apoptosis in glioblastoma cells [67]. The activation of this pathway presents a potential immunotherapeutic candidate for glioblastoma treatment.

### 4.3. Antioxidant Activity

Reactive oxygen species (ROS), which encompass oxygen free radicals, are known to not only damage cellular components but also play a role in cellular differentiation, signal transduction, and the induction of apoptosis and necrosis, thereby contributing to the natural aging process. Recent research has highlighted the antioxidant properties of HEPs as a significant area of investigation. Studies have demonstrated that the ability of HEPs to scavenge hydroxyl radicals increases with higher concentrations of polysaccharides, suggesting their potential as natural antioxidants [15]. Experiments were performed employing ABTS, DPPH, and hydroxyl radical scavenging assays. The results indicated that within a defined concentration range, the ability of *Hericium erinaceus β-glucan* to scavenge ABTS radicals exhibited a positive correlation with increasing concentrations [20]. Notably, at a concentration of 10 mg/mL, *Hericium erinaceus β*-glucan exhibited the highest scavenging rates for both DPPH and hydroxyl radicals. Furthermore, HEPs were found to inhibit apoptosis and ROS production induced by the fusarium toxin deoxynivalenol, with fluorescence measurements using DCFH-DA indicating a significant reduction in intracellular ROS levels, thereby reaffirming the antioxidant activity of HEPs [17,43]. Additionally, it was reported that HEP residues also demonstrated substantial antioxidant and anti-aging properties, providing protective effects on hepatic and cerebral tissues, which could be developed into functional foods and nutritional supplements aimed at preventing aging and age-related diseases [40].

Oxidative stress is a critical factor in the pathogenesis of various diseases, including those affecting the nervous system, liver, diabetes, obesity, and other conditions, and it plays a vital role in the aging process and inflammatory diseases [68]. Research has indicated that antioxidants confer protective effects on the liver. In experiments assessing the liver’s antioxidant capacity, Wu et al. [58] observed a significant increase in T-AOC in liver tissues from the HEP 500 and HEP 750 groups, alongside a notable reduction in MDA levels in the HEP 500 group compared with the controls, although no significant effects were noted for the liver’s GSH-PX and T-SOD activities. It was further demonstrated that HEPs significantly enhanced the activities of T-AOC, SOD, GSH-PX, and CAT in murine liver tissues while concurrently reducing MDA levels, thereby effectively protecting the liver from oxidative damage and partially restoring its metabolic function [34].

Moreover, the antioxidant activity of HEPs has been shown to be significant in gastrointestinal diseases. It was found that HEFPs could diminish MDA production, enhance SOD and CAT activities, and mitigate oxidative damage associated with ulcerative colitis (UC) [26]. The study examined the effects of HMP and HFP polysaccharides, which were observed to elevate the activities of SOD, CAT, GSH, and ATP. This enhancement contributes to the improved resistance and reparative capabilities of gastric tissues in response to mucosal injury [56]. Additionally, HEPs were shown to inhibit free radical production, alleviate mitochondrial dysfunction, and preserve mitochondrial integrity and function, thus preventing gastric cell damage due to oxidative stress [53].

Simultaneously, HEPs also exert antioxidant effects in immune organs. It was reported that HEPs significantly reduced the MDA levels in the thymus and spleen while markedly increasing the activities of GSH-PX and T-AOC in the immune organs of mice [62].

### 4.4. Anti-Inflammatory Activity

Inflammation represents a physiological response of the host, serving as a protective mechanism against pathogens (such as bacteria and viruses), trauma, and various diseases. However, when inflammation becomes chronic and leads to an excessive accumulation of immune cells, it can disrupt the immune system and impair tissue function. Numerous prevalent diseases, including Alzheimer’s disease, cancer, arthritis, asthma, gout, multiple sclerosis, diabetes, and depression, are linked to inflammatory processes. Therefore, following the resolution of an infection, it is crucial to mitigate the exacerbation of the inflammatory state to facilitate the restoration of tissue homeostasis.

Research has shown that HEP-1, which is derived from HEFB, alleviates liver damage and inflammatory responses associated with HFD and streptozotocin (STZ) in murine models. Additionally, HEP-1 has been found to decrease both the accumulation and size of lipid droplets within the liver [52]. It was reported that, in comparison with the control group, the treatment group receiving HEP exhibited decreased serum levels of LPS, TLR4, MyD88, and NF-κB, alongside a significant reduction in the gene expression of IL-1β and IL-6. These findings suggest a reduction in liver inflammation and apoptosis in hens suffering from nonalcoholic fatty liver disease. Additionally, the HEP treatment group displayed significantly lower AST, TG, LDL-C levels, liver index values, and abdominal fat rates, indicating an improvement in hepatic steatosis. Collectively, these results suggest that HEPs exert a regulatory effect on lipid metabolism, thereby enhancing liver function [25].

In pathological conditions, the excessive release of inflammatory mediators elevates oxidative stress levels, which in turn exacerbates the inflammatory response [69]. The administration of polysaccharides derived from HEM has been shown to inhibit the reduction of SOD enzyme activity and improve the free radical scavenging ability of Caco-2 cells. This intervention effectively mitigates oxidative stress and leads to a significant decrease in inflammatory markers, such as TNF-α, IL-6, and IL-8, in the serum of rats. Furthermore, following oral administration of HEPs, the morphological abnormalities of mitochondria in the intestinal epithelial cells of rats with UC were ameliorated, including the swelling and rupture of mitochondrial membranes [48]. These findings underscore the significant role of polysaccharides from HEM in addressing inflammation-induced oxidative stress and mitochondrial dysfunction, while also providing insights into the further development and application of *Hericium erinaceus* in functional foods and adjunctive therapies.

### 4.5. Intestinal Health Improvement Activity

**Figure 5:**
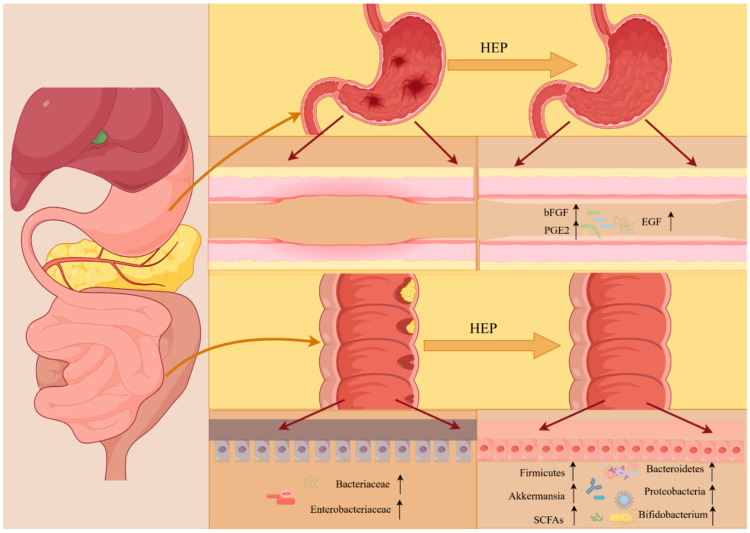
Schematic diagram of HEPs’ potential for enhancing gastric protection and promoting intestinal health, “↑” = increase.

The host gastrointestinal tract harbors a diverse array of microorganisms that significantly influence both health and the pathophysiological processes of the intestine, as shown in Figure 5. Fungal polysaccharides, particularly *Hericium erinaceus β*-glucan, have been identified as crucial contributors to the enhancement of human intestinal microbiota [70]. Research conducted by Cui demonstrated that HEP-1, derived from HEFB, can augment the relative abundance of beneficial genera such as *Akkermansiella*, *Dunaliella*, and *Lactobacillus*, thereby modulating the composition and abundance of intestinal microorganisms to sustain the homeostasis of the gut microbiome [52]. Investigations by Ren et al. on low molecular weight HEPs revealed their capacity to reverse nearly all bacterial species enhanced by DSS, particularly *Akkermansia mucilaginosa*, while also promoting the transformation of intestinal flora, exhibiting a pronounced prebiotic effect [55]. Additionally, experimental findings from the oral administration of polysaccharides extracted from HEFB indicated that HEPs could serve as a novel dietary supplement and prebiotic for the amelioration of colitis [38].

Moreover, SCFAs are pivotal in maintaining the normal physiological function of the intestine, as well as the morphology and functionality of colonic epithelial cells. A significant increase in SCFA concentration was observed in the feces of male and female participants treated with HEPs, alongside an elevation in beneficial bacterial populations such as *Bifidobacterium*, *Fecalibacterium*, *Blautia*, *Butyrococcus*, and *Lactobacillus*. Concurrently, a notable reduction in pH level and inhibition of the relative abundance of pathogenic genera such as *Escherichia coli-Shigella*, *Klebsiella*, and *Enterobacter* were recorded, which influenced microbial enzymatic activity [18]. These findings were substantiated by evidence indicating that two polysaccharides derived from HEFB have the potential to enhance the diversity and richness of microbial communities, elevate SCFA levels, and foster intestinal stability [21].

Given that intestinal microbial metabolites are produced in proximity to the intestinal epithelium, they exert a substantial influence on intestinal barrier function and immune responses [71]. The anti-ulcerative properties of HEM polysaccharide (EP-1) were assessed through a rat model of acetic acid-induced ulcerative colitis. The findings indicated that EP-1 effectively mitigated congestion and erosion of the colonic epithelial mucosa, modulated the intestinal microbiota, and increased the levels of SCFAs [47]. Furthermore, cynomolgus monkeys with ulcerative colitis exhibited reduced diarrhea, weight gain, enhanced intestinal function, and a restructured intestinal microbiome through a non-artificially induced model that closely resembles human ulcerative colitis [72]. Additionally, it was confirmed that HEPs effectively mitigate intestinal mucosal injury, thereby preserving the integrity of the intestinal mucosal structure, increasing the expression of intestinal tight junction proteins, repairing damaged epithelial tight junctions, and reducing intestinal permeability, collectively promoting intestinal health [34]. The experiments also indicated that HEPs significantly increased the abundance of *Lactobacillus* species and activated the AhR, upregulating the mRNA expression levels of its target genes CYP 1A 2 and IL-22 to bolster the intestinal barrier, thus maintaining the integrity of intestinal function [25].

Furthermore, studies have indicated that *Hericium erinaceus* can enhance the immune response and anti-obesity potential of intestinal flora [73]. In conclusion, these findings suggest that *Hericium erinaceus* holds promise for further development as a prebiotic, establishing a foundation for the advancement of HEPs in dietary supplements and health food additives.

### 4.6. Gastric Protective Activity

Gastrointestinal health serves as a critical indicator of overall human health. A significant number of individuals worldwide continue to experience gastric ulcers, which impose considerable health burdens on both individuals and society [74]. *Hericium erinaceus*, a traditional edible fungus, is recognized for its medicinal properties and its potential to ameliorate gastrointestinal disorders, as shown in Figure 5. The *β*-glucan H6PC20 and *α*-heteropolysaccharide HPB-3 derived from HEFB exhibit notable protective effects on the gastric mucosal epithelium in rats. This protective effect is characterized by the maintenance of a smooth gastric mucosa, the absence of significant glandular structural disorders, reduced mucosal congestion, and diminished inflammatory cell infiltration, as well as a reduction in the area of gastric ulcer formation. Furthermore, these compounds appear to influence the expression levels of EGF, which is beneficial for enhancing mucosal blood flow and promoting epithelial cell proliferation, thereby facilitating tissue healing. Additional investigations have indicated that the macromolecular dextran H6PC20 may mitigate gastric mucosal injury by attenuating the inflammatory response, decreasing oxidative damage, and enhancing the secretion of protective and reparative factors such as EGF, bFGF, and PGE2 within the gastric mucosa. In contrast, the small molecule heteropolysaccharide HPB-3 may confer protection to the gastric mucosa through its anti-inflammatory properties [75]. The study revealed that both HMP and HFP provided protective benefits to the gastric mucosa by enhancing cell migration, with HMP showing greater effectiveness in animal models of acute gastric ulcers [56]. Moreover, cellular experiments have demonstrated that the gastric protective properties of HMP are predominantly linked to its triple helix configuration, which exhibits a markedly greater efficacy compared with its degradation products. The incorporation of nano-silica (nSiO2) was found to enhance the gastric protective efficacy of HMP [76].

### 4.7. Neuroprotective Activity

*Hericium erinaceus* exhibits significant therapeutic potential for neurodegenerative disorders characterized by neuronal dysfunction, as it has been shown to enhance cognitive abilities and safeguard nerve cells from apoptosis [77,78]. The compound amyloban, derived from *Hericium erinaceus* extract, has been reported to mitigate elevated levels and the turnover of dopamine metabolites, inhibit excessive activation of the dopaminergic system in the hip joint, and consequently ameliorate social deficits in mice subjected to social failure stress [79]. Research indicates that a compound present in HEM possesses neuroprotective properties and demonstrates therapeutic efficacy against conditions such as depression, ischemic brain injury, Alzheimer’s disease, and Parkinson’s disease [80].

Preliminary investigations have revealed that HEM exerts a protective effect against neurodegeneration induced by a high-fat diet and HFSD. The underlying mechanism appears to involve the downregulation of mRNA expression for TNF-*α* and IL-1*β* in murine models, alongside an upregulation of NGF and NeuN in the hippocampus. This modulation contributes to improved spatial learning, reduced neuronal loss, and alleviation of abdominal fat and metabolic irregularities [81].

Various bioactive constituents of *Hericium erinaceus* have been identified as neuroprotective agents. For instance, polysaccharides extracted from HEM could enhance learning, cognition, and memory in behavioral assessments, while also confirming that the polysaccharide PHEB alleviates symptoms associated with Alzheimer’s disease. The primary mechanism of action for PHEB involves the inhibition of brain injury, reduction of amyloid beta deposition, and prevention of tau hyperphosphorylation, which collectively lead to increased expression of cholinergic factors and modulation of the Akt/SHANK3/mTOR signaling pathway. Additionally, it addresses oxidative stress-related calcium homeostasis through the phosphorylation of CaMK II/IV [37].

### 4.8. Antiviral Activity

HEPs demonstrated a significant inhibitory effect on viral replication [82]. Research conducted by various scholars has demonstrated that HEPs have the capacity to ameliorate intestinal damage in Muscovy ducklings resulting from MDRV infection. Furthermore, HEPs were found to stimulate the lymphocyte homing mechanism and enhance the expression levels of various genes, adhesion molecules (*α*4*β*7 and LFA-1), and chemokine receptors (CCR7, CCR9, and CCR10) associated with cellular activities, including protein translation, cytokine interactions, and adhesion molecule functions [83]. Additionally, HEPs effectively mitigated morphological alterations in key immune organs such as the liver, spleen, and thymus in mice infected with MDRV. This treatment also improved antioxidant functions, serum protein levels, antibody levels, and complement levels, thereby reducing damage to immune and liver cells and enhancing the overall immune response. Moreover, HEPs were shown to inhibit apoptosis in Muscovy ducklings infected with MDRV [84].

### 4.9. Other Effects

Ovarian Protective Activity: HEPs significantly enhanced the quantity of classified follicles, namely SYFs, as well as the total follicle count in hens. Concurrently, the levels of LH, E2, and FSH in the HEP treatment group were markedly elevated compared with the control group, suggesting that HEPs substantially increase serum reproductive hormone concentrations [58].

Hypoglycemic Activity: HEP-1, a low molecular weight polysaccharide derived from HEFB, significantly improved glucose tolerance in murine models, reduced blood glucose levels, and modulated the IRS/PI3K/AKT signaling pathway. This resulted in decreased insulin resistance and enhanced glucose uptake and glycogen synthesis in hepatocytes [52].

Anti-Apoptotic Protective Activity: HEPs exhibited a pronounced inhibitory effect on apoptosis induced by hydrogen peroxide. The study further established that HEPs could suppress the death receptor pathway and caspase-8 activation by downregulating the expression of TNFR1, Fas, and FasL. Additionally, HEPs promoted the expression of Bcl-2 while decreasing the levels of Bax and Bad, thereby regulating the endogenous mitochondrial pathway and influencing caspase-9 and Cyt-C, which collectively indicated a protective effect against cell apoptosis [17].

Anti-Aging Activity: Polysaccharides extracted from *Hericium erinaceus* could extend the lifespan of wild-type yeast cells (CLSs) and significantly enhance cell survival rates under heat shock conditions at 51 °C, thereby indicating potential anti-aging properties [35].

Moreover, additional studies have indicated that the hot water extract of *Hericium erinaceus* possesses a degree of antiplatelet and anticoagulant activity in vitro, which may provide a foundation for further investigation into the bioactivity of its polysaccharides [85].

## 5. Toxicity

A diverse array of heteroagglutinins present in HEFB has demonstrated cytotoxic effects on various cell lines, characterized by diminished cell viability and suppressed cell proliferation [86]. Consequently, it is imperative to investigate the toxicity associated with HEPs. Research indicates that at concentrations below 800 μg/mL, HEPs do not exhibit any inhibitory effects on the viability of the RAW264.7 macrophage cell line [16]. Furthermore, it was reported that neither HMP nor HFP exhibited cytotoxic effects on gastric epithelial cells at a concentration of 500 μg/mL [56]. Additionally, the viability of RAW 264.7 cells was evaluated utilizing an MTT assay, which indicated that low molecular weight heparin exhibited negligible toxic effects on murine macrophages [55].

Moreover, HEPs not only lacked toxicity toward cells but also promoted cell proliferation following treatment. The proliferation of cells treated with HEPs was significantly greater compared with the untreated control group [17]. Concurrently, HEPs can mitigate the toxicity of *α*-synuclein, decrease reactive oxygen species (ROS) levels, enhance mitochondrial function, exhibit anti-aggregation properties, and contribute to the extension of cell lifespans [35]. Therefore, despite the presence of various cytotoxic effects associated with HEFB, the toxicity of its polysaccharide components appears to be minimal.

## 6. Applications

### 6.1. Application in the Food Industry

*Hericium erinaceus*, commonly known as lion’s mane mushroom, is esteemed for its culinary attributes, nutritional benefits, and medicinal properties. It has been incorporated into healthier flatbreads, utilizing *Hericium erinaceus* powder and mycelium in place of traditional wheat flour [87,88]. Furthermore, the combination of lion’s mane and pink oyster mushrooms is noted for its high protein content, amino acids, dietary fibers, *β*-glucans, and free soluble sugars, contributing to its superior nutritional profile. A vegetarian shrimp alternative made from these mushrooms has garnered significant interest in the market [24].

Polysaccharides derived from HEFB, when fermented with *Lactobacillus gasseri* JM1, exhibited a more pronounced protective effect against acrylamide-induced cellular damage compared with polysaccharides from unfermented fruiting bodies. This fermentation process was shown to enhance transepithelial electrical resistance (TEER), reduce cellular permeability, and more effectively mitigate damage to the intestinal barrier [31]. Additionally, semi-solid enzyme fermentation altered the physical and chemical properties of HEFB powder and polysaccharides, thereby improving their protective effects against gastric mucosal cell injury, increasing the inhibition rate of gastric ulcers, enhancing resistance to oxidative stress, and regulating intestinal flora [30]. This has led to the development of solid beverages aimed at improving intestinal function, as well as popular fermented products such as *Hericium erinaceus* wine and vinegar, which highlight the potential of HEP fermentation products [9,11]. The fermentation process not only enhances the biological activity and nutritional value of *Hericium erinaceus* products but also improves their flavor and market competitiveness, while positively impacting health and environmental sustainability [89]. Additionally, *Hericium erinaceus* tea is recognized for its rich array of active biological components and is favored as a beverage, alongside coffee blends that include *Cordyceps militaris* and *Hericium erinaceus* [4].

In addition, the waste residue after water extraction of polysaccharides from edible fungi accounts for a large proportion, which contains a large amount of water-insoluble dietary fiber composed of *β*-glucan, chitin, hemicellulose, and mannan. Studies have shown that it has a certain inhibitory effect on the digestion of starch and fat, such as the currently popular meal replacement powder containing HEPs [9,90]. The extracted residue is treated as fertilizer or solid waste, which not only pollutes the environment but also wastes resources. The by-products of edible fungi such as *Hericium erinaceus* have good physical and chemical properties and physiological functions and are also used as an ideal raw material for industrial production of edible fungi dietary fiber.

### 6.2. Application in the Health Industry

HEPs exhibit significant prebiotic properties, which can modulate the composition and metabolic functions of the gastrointestinal microbiota, thereby contributing to nutritional equilibrium and disease prevention. These characteristics position HEPs as a promising candidate for development into edible dietary fibers and prebiotic-related health products, an area of considerable interest within nutritional science research [91]. For instance, *Lactobacillus* fermentum HP3-mediated *Hericium erinaceus* juice has been identified as a health-promoting supplement with potential applications in diabetes management [87]. Research indicates that fungi, including *Hericium erinaceus*, are rich in bioactive compounds such as polysaccharides and ergothioneine, which are associated with longevity benefits. This provides a substantial foundation for the creation of anti-aging and longevity-focused health products, such as medicinal wines derived from *Hericium erinaceus* [11,92]. Furthermore, studies have demonstrated that *Hericium erinaceus* can enhance cognitive performance in healthy young individuals, alleviate emotional distress, and diminish perceived stress levels. For example, the administration of oral supplements derived from HEFB offers a theoretical framework for investigating the role of HEPs in the domain of brain health foods [9,93].

### 6.3. Application in Drug Delivery Systems

Polysaccharides are increasingly recognized as optimal raw materials for the development of drug delivery systems. HEPs have been utilized as immune enhancers to augment immune responses. The application of HEPs in the form of nanoparticles has been shown to significantly prolong the PCV2-specific IgG immune response and elevate cytokine levels, thereby demonstrating enhanced immunological benefits and facilitating macrophage maturation [94].

Chitosan, a natural polymer, exhibits favorable properties such as biocompatibility, non-toxicity, and biodegradability, making it a widely employed material for drug delivery [95]. A novel drug delivery system was engineered through the cross-linking of heparin (HEP) with chitosan and multi-walled carbon nanotubes (CS-MWCNT-HEP) to improve drug absorption. The in vivo distribution of the drug was evaluated visually using fluorescence imaging of Cy7 in mice that received free Cy7, Cy7-HEP, Cy7-CS-MWCNT, and Cy7-CS-MWCNT-HEP formulations [96].

Two distinct types of stable and well-characterized nanoparticles with negatively charged surfaces were synthesized: selenium-HEP-PLGA (Se-HEP-PLGA) nanoparticles and selenium-modified HEP-PLGA (HEP-PLGA-Se) nanoparticles. Both formulations demonstrated a significant enhancement in macrophage phagocytic activity and elevated the expression levels of nitric oxide (NO) as well as several cytokines, including TNF-*α*, IL-1*β*, and IL-6 [97].

Furthermore, resveratrol (RES), *β*-glucan-chitosan nanoparticles (DS-CS NPs), and resveratrol-loaded sulfated *β*-glucan-chitosan nanoparticles (DS-CS-RES NPs) could inhibit the release of NO, as well as the protein levels of NF-κB, p65, STAT 1, and TLR 4, along with the secretion of inflammatory mediators such as TNF-*α*, IL-6, and IL-1*β* in LPS-induced RAW 264.7 macrophages. The findings indicated that the anti-inflammatory efficacy of DS-CS-RES NPs containing resveratrol surpassed that of DS-CS, NPs and RES, suggesting a potential synergistic anti-inflammatory effect between DS-CS nanoparticles and RES. Additionally, the encapsulation of hydrophobic RES within hydrophilic DS-CS nanoparticles enhances cellular absorption and bioavailability [98]. This research provides a novel perspective on utilizing *Hericium erinaceus β*-glucan-chitosan as a functional carrier.

### 6.4. Application in the Field of Biomaterials

Natural fungal *β*-glucans typically exhibit distinctive helical or highly branched structures, which confer a range of biological activities, including immune modulation and anti-inflammatory properties [99,100,101]. The *β*-glucan derived from *Hericium erinaceus*, characterized by its helical structure and high viscosity, forms a physical entanglement with the bioactive compound tannic acid. The resultant *Hericium erinaceus β*-glucan/tannic acid (HEBG/TA) hydrogel demonstrates significant anti-inflammatory and antioxidant activities, as well as capabilities in hemostasis, neovascularization, and tissue regeneration. This research underscores the potential of integrating natural *β*-glucans with bioactive agents for applications in skin repair [102].

Moreover, the by-products of *Hericium erinaceus* residue (HER) and pineapple peel (PP) serve as valuable sources of cellulose and chitosan. Chitosan is noted for its excellent hygroscopic properties and moisture retention, making it suitable for gas exchange applications, particularly in medical dressings for wound management and healing, thus emerging as a promising biomaterial [103]. Additionally, utilizing a freeze-thaw technique, chitin was solubilized in an aqueous solution of sodium hydroxide and urea, followed by its regeneration into a hydrogel [104]. A novel, pH-sensitive composite hydrogel was synthesized via a Schiff base reaction involving the aldehyde groups of oxidized hydroxyethyl cellulose (OHEC) and the amino groups of hydroxymethyl chitosan (CMCS). This composite hydrogel demonstrated advantageous swelling characteristics and responsiveness to pH variations. The clear and stable three-dimensional network structure of this hydrogel facilitates drug loading and release, indicating its potential as a carrier in drug delivery systems [105]. Further investigations have revealed the feasibility of producing electric field-sensitive hydrogels, magnetic chitin hydrogels from *Hericium erinaceus* residue, and magnetic chitin/Cu hydrogel nanocomposites utilizing modified pineapple peel, cellulose, chitosan from *Hericium erinaceus*, and gelatin [106,107,108]. A novel magnetic hydrogel composite was also developed using chitin/polyvinyl alcohol (PVA) as the carrier [109]. In other applications, chitin extracted from *Hericium erinaceus* has shown promising prospects. Carboxymethylation of this chitin was performed to create carboxymethyl chitin hydrogels with varying degrees of substitution, revealing that a higher degree of substitution correlated with an increased swelling capacity, enhanced pH sensitivity, and selective adsorption of various dyes [110].

### 6.5. Other Applications

Plant polysaccharides have shown broad application prospects in various fields due to their diversity of biological activities [111,112]. *Hericium erinaceus* is recognized not only for its culinary and medicinal value but also for its abundance of bioactive compounds, which confer a range of advantageous physiological effects on the human body. In addition to polysaccharides, *Hericium erinaceus* also contains a variety of molecules and other bioactive components, which cooperate with polysaccharides to enhance their overall biological activity [1]. In the cosmetic industry, HEPs have been utilized in the formulation of skin and hair care products, attributable to their antioxidant and anti-inflammatory properties. These polysaccharides play a crucial role in safeguarding the skin against oxidative stress caused by free radicals and mitigating inflammation, thus contributing to retardation of the skin aging process. Consequently, the beneficial attributes of HEPs render them a significant ingredient in various cosmetic formulations, including face creams, lotions, essences, and other related products [113].

## 7. Discussion

*Hericium erinaceus*, a valuable herbal medicine in traditional Chinese medicine, is recognized for its dual role as both a medicinal and nutritional substance. This review examines various aspects of HEPs, including their extraction, separation, purification, physical and chemical properties, structural characteristics, biological activities, toxicity, and applications. The predominant method for extracting HEPs is the water extraction technique. Analysis of monosaccharide compositions revealed that galactose, glucose, mannose, and fucose are the most frequently occurring sugars in HEPs. These polysaccharides exhibit a wide range of biological activities, such as immune modulation, anti-tumor effects, antioxidant properties, anti-inflammatory actions, enhancement of intestinal health, gastric protection, antiviral effects, anti-aging benefits, and hypoglycemic activity. Despite the advancements made in various research domains, numerous opportunities and challenges remain. Firstly, the large molecular weight and complex structure of HEPs present limitations in current structural characterization methods, necessitating the development of novel and direct approaches for such characterization. Secondly, the extraction conditions, including the temperature, duration, and solid-to-liquid ratio, significantly influence HEPs’ properties. However, systematic investigations into the relationships between these conditions and the structure, solubility, type, and pharmacological activities of HEPs are still lacking. Thus, establishing a comprehensive system linking extraction methods, effective components, and pharmacological activities is essential. Thirdly, while research on HEPs has progressed, there is insufficient exploration of their novel pharmacological activities, warranting further investigation into their potential and mechanisms of action. Fourthly, the quality of an HEP is intrinsically linked to its pharmacological efficacy, highlighting the need for further studies aimed at enhancing its biological activity. Finally, the application of HEPs in the food sector presents extensive opportunities, and the research on their toxicity still needs further in-depth analysis and exploration to ensure their safety and reliability. It is anticipated that the findings presented in this review will serve as a valuable reference for future research on HEPs.

## Figures and Tables

**Table 3:** The biological activities and mechanisms of *Hericium erinaceus* polysaccharides.

Polysaccharide Name	Biological Activity	Experimental Subject	Effects and Mechanisms	Reference
HECP, HERP	Antioxidant activity	Male adult Sprague Dawley rats	Enhancing the activities of SOD and GSH-PX in gastric tissues of gastric ulcer rats; reducing the content of MDA.	[50]
HEP	Antioxidant activity	In vitro	The scavenging ability of *Hericium erinaceus* polysaccharide on hydroxyl radical increased with an increase in the polysaccharide concentration.	[15]
Hep-2	Antioxidant activity	GES-1 cell	Producing T-SOD and GSH-px in a concentration-responsive manner to protect cells from oxidative damage.	[46]
HEP	Anti-inflammatory activity	Jingbai laying hens	Reduced inflammatory cell infiltration; alleviated inflammation; exhibited fewer or smaller cytoplasmic lipid vacuoles.	[58]
wHEP-1, wHEP-2, wHEP-3	Anti-inflammatory activity	Half male Sprague Dawley rats	Improvements in mucosal degeneration and necrosis, cryptitis and crypt dilation, and mucosal and submucosal neutrophilic and monocytic infiltration.	[39]
wHEP-1	Anti-inflammatory activity	Caco-2 cell	The protective effect on epithelial cells induced by LPS through the TLR4/NF-κB pathway is significant.	[44]
HEFPs	Intestinal health improvement activity	Male C57BL/6J mice	Enhancing the richness of the gut microbiota, which is composed of Firmicutes, Bacteroidetes, Verrucomicrobia, Proteobacteria, Actinobacteria, and a few other microorganisms.	[26]
HEP	Intestinal health improvement activity	Male specific pathogen-free ICR mice	Enhancing the species richness and diversity of the gut microbiota; significantly increasing the expression levels of SCFA receptors; regulating and restoring imbalance in the gut microbiota.	[33]
HEPs	Intestinal health improvement activity	Fresh feces and intestinal contents (small intestine, cecum, and colon)	Significantly increasing the relative abundance of Ruminococcaceae and Akkermansiales in the gut microbiota.	[38]
HECP, HERP	Gastric protective activity	Male Sprague Dawley rats	Reducing the ulcer area in gastric tissue; improving mucosal structure; gastric gland structure tending to be complete; alleviating the inflammatory response of gastric mucosa in ulcerative rats; increasing the release of gastric defensive factors; promoting the regeneration of gastric mucosa.	[50]

## Data Availability

No new data were created or analyzed in this study. Data sharing is not applicable to this article.
